# Tissue engineering strategies for the induction of angiogenesis using biomaterials

**DOI:** 10.1186/s13036-018-0133-4

**Published:** 2018-12-27

**Authors:** Shirin Saberianpour, Morteza Heidarzadeh, Mohammad Hossein Geranmayeh, Hossein Hosseinkhani, Reza Rahbarghazi, Mohammad Nouri

**Affiliations:** 1 Stem Cell Research Center, Tabriz University of Medical Sciences, Imam Reza St., Golgasht St, Tabriz, 5166614756 Iran; 2 Department of Molecular Medicine, Faculty of Advanced Medical Sciences, Tabriz University of Medical Sciences, Tabriz, Iran; 3 Neuroscience Research Center, Imam Reza Medical Center, Tabriz University of Medical Sciences, Tabriz, Iran; 4 Innovation Center for Advanced Technology, Matrix, Inc., New York, NY 10029 USA; 5 Department of Applied Cell Sciences, Faculty of Advanced Medical Sciences, Tabriz University of Medical Sciences, Tabriz, Iran

**Keywords:** Scaffolds, Vascular regeneration, Cell source, Genetic and proteomic manipulation, Delivery methods

## Abstract

Angiogenesis is touted as a fundamental procedure in the regeneration and restoration of different tissues. The induction of de novo blood vessels seems to be vital to yield a successful cell transplantation rate loaded on various scaffolds. Scaffolds are natural or artificial substances that are considered as one of the means for delivering, aligning, maintaining cell connection in a favor of angiogenesis. In addition to the potential role of distinct scaffold type on vascularization, the application of some strategies such as genetic manipulation, and conjugation of pro-angiogenic factors could intensify angiogenesis potential. In the current review, we focused on the status of numerous scaffolds applicable in the field of vascular biology. Also, different strategies and priming approaches useful for the induction of pro-angiogenic signaling pathways were highlighted.

## Introduction

With regard to the function of many types of cells in restoring tissue performance, regenerative medicine with palliative treatments would be considered as an alternative medicine for the replacement or regeneration of various tissues and organs. Regenerative medicine uses different technologies and methodologies including; tissue engineering techniques, cell transplantation approach, stem cell biology, biomechanics, prosthetics, and nanotechnology [1]. By using appropriate physical substrates and the induction of cellular signaling pathways, these novel approaches provide the basic interaction and essential integration of plated cells with underlying biomaterials scaffolds and crosstalk with the neighboring cells. Up to the present, diverse methodologies and approaches have been found in this era (Table 1). In the construction of tissue-engineered grafts, it seems that the promotion of vascularization and angiogenesis is a fundamental step for efficient organ reconstitution and replacement [2]. The progression and development of blood vessels into the transplanted tissues are stimulated following induction of pro-angiogenic signaling pathways. In line with this statement, controlling the angiogenic switch and vessel development is essential for the normal activity of transplanted cells and/or acquisition of novel phenotypes. Angiogenesis status is determined by the balance between pro- and anti-angiogenesis factors and cytokines [3]. It has been determined that the in situ production of pro-angiogenic factors promotes the vascular regeneration in response to tissue demands [4]. Early-stage angiogenesis is promoted due to the secretion of most important factors VEGF, bFGF, Ang-2 and other ligands by different cells located in the close proximity to target sites and remote areas. Following the angiogenic switch, the expression of receptor tyrosine kinases such as VEGFR-2 and Tie-2 along with Tie-1 is up-regulated on ECs surfaces thereby promotes intracellular signaling pathways [5]. After the induction of ECs by pro-angiogenic factors, the cell-to-cell connection is weakened which followed by degradation of basal membrane governed by the activation of MMP-2 and -9 [6]. Activated ECs proliferate and migrate in response to the concentration gradient of pro-angiogenic factors. Two EC types are phenotypically detectable based on the cell surface markers; tip cells, CD34, and CD31 positive cells, that are located at the sprout tips and characterized by the existence of filopodial extensions and stalk ECs, CD31 positive and CD34 negative cells, constitute the lumen of nascent vessels [7]. To stabilize the vessel structure, the attachment of Ang-1 to cognate receptor Tie-2 increases the integration of ECs with neighboring cells and surrounding peri-vascular pericytes thereby promoting vascular maturation and reducing migration activity of ECs. In addition to angiogenesis initiated by sprouting mechanism, other alternative remodeling mechanisms such as intussusception and bridging were also described as inverted angiogenesis in the context of vascular structure [8]. Intussusception is touted as trans-vascular tissue pillars formed inside vessels lumen extensively seen in developing vessels to form multi-vascular branches. In bridging vascular remodeling, intraluminal endothelial bridges are formed by invagination of the basal membrane while incorporating polarized ECs with simultaneous cytoskeletal adaptation from both sides to each other thereby dividing the luminal space into multi-vascular units [9]. It is well known that the ECM composition, stiffness could affect ECs functional behavior, differentiation, and network formation properties. Alteration of ECM consistency and substrate composition caused to ECs lose tubulogenesis capacity and changes migration activity. Mechanical stimuli can affect the expression of genes participating in angiogenesis signaling pathways. After cell adaptation to mechanical forces induced by surrounding environment, the emergence of internal and external forces dictates location and shape of organelles and biomolecules and their interaction with cell cytoskeleton, resulting in the adaptation of biochemical responses and angiogenesis modulation [10]. Controllable angiogenesis induction will enable us to increase the final extent of transplanted tissue to host tissues. This review article familiarizes the readers with the different scaffolding biomaterials that have been used for the restoration of vascular structure in a different milieu and novel approaches applicable to harness the angiogenic potential of biomaterials in different contexts.

**Table 1:** Progress in the field of regenerative medicine

Finding/Experiment	Ref.
First cell transplantation: Bone marrow transplant (1968)	[116]
Discovery of stem cells in human cord blood (1978)	[117]
First engineered tissue transplantation: skin (1981)	[118]
First in vitro stem cell line developed from mice (1981)	[119]
First engineered vessel structure was synthesized (1986)	[120]
Adult stem cells were used for vascular regeneration by Asahara (1997)	[121]
Isolation of human embryonic stem cells (1998)	[122]
First laboratory-grown organ: an artificial bladder implanted in a patient suffering from myelomeningocele (1999)	[123]
Implantation of first engineered tubular organs (urine conduits) (2004)	[124]
Discovery of stem cells derived from amniotic fluid and placenta (2007)	[125]
First solid organ engineered by recycling donor liver (2009)	[123]
3D-printed vascular networks direct therapeutic angiogenesis in ischemic condition (2017)	[126]

### Vasculogenesis and angiogenesis; terminology and definitions

There are two fundamental primary mechanisms implement the formation of new blood vessels; vasculogenesis and angiogenesis [11]. The formation of fetus heart and primary vascular network from yolk sac is governed by vasculogenesis while angiogenesis mainly participates in vascular remodeling post-natal period [11]. In response to cytokines gradient, EPCs recruit to target sites and participate to restore luminal continuity [12]. The critical role of EPCs has been documented during fetal growth and development [13]. Angioblasts are the primary source of ECs at the early-stage development of fetus with a great capacity to differentiate into functional ECs. These cells form clusters to generate tube-like structures which are further supported by cells expressing α-actin namely α-SMCs [14–16]. Cells expressing α-SMC maintains vascular integrity and the tight junction with ECs at the luminal surface by the synthesis of collagen and ECM substrates such as elastin. These fibers give an opportunity for vessels to preserve contractility and increase mechanical resistance [17]. To induce the generation of de novo blood vessels, proteases degrade the ECM at the site of angiogenesis [18, 19]. During the promotion of an active angiogenesis status, vessel branching occurs in three distinct stages as follows; quiescence, activation, and resolution. In the stage of quiescence, cell proliferation is inhibited when ECs are in close contact with VE-cadherins from neighboring cells. The persistent interactions are intensified by the activity of surrounding pericytes. Following the angiogenic switch, for example, the production of angiogenesis factors such as VEGF, it facilitates pericytes detachment from the basement membrane and therefore ECs have enough space to migrate to the target sites. The accelerated degradation of basement membrane paves the ground for extending ECs to migrate [20]. Considering the important role of blood vessels in the nourishment of various cells, it is believed that providing novel techniques to promote large-scale angiogenesis with distinct growth factor are key factors for successful engineering of large organs [21, 22].

### The application of cells for the induction of angiogenesis

The application of SCs for vascular regeneration and the existence of pro-vasculogenic EPC subpopulation have been extensively used in recent clinical trials [23]. These cells have per se potential to modulate the function of blood vessels in different trials. In support of this statement, scientists have strived to exploit the pro-angiogenic ability of EPCs for treating myocardial infarction, ischemic changes, and peripheral vascular disease and wound healing as well [20, 24]. After initiation of ischemic changes, there is an urgent need for the pro-angiogenic activity of transplanted cells on engineered vascular constructs while inhibiting angiostatic pathways [25]. The advent of novel approaches must be able to afford the pitfalls and drawbacks correlated with classical therapeutic methods. For instance, the most prevalent clinical solution for heart attack is the replacement of injured vessels with autologous vein and arteries to restore blood supplementation [26]. Considering disease management and pathological changes associated with age, bulks of patients without normal vessels are candidates for the vessels grafting. For example, the lack of inappropriate angiogenesis rate contributes to non-completed cardiac tissue restoration caused by ischemia and hypoxia. Therefore, an essential clinical management is required to efficiently perfuse blood to the ischemic sites through the promotion of angiogenesis by engaging pro-angiogenic effectors. Noteworthy, the induction of vessels formation from pre-existing vasculature bed and recruitment of EPCs and CECs could be an appropriate strategy in favor of vasculogenesis [27, 28]. Along with the control of angiogenesis signaling pathway and the dynamics of participant cells, fabrication of physiological microenvironment via a plethora of semi-synthetic and synthetic scaffolds similar to in vivo condition is inevitable. In this regard, the application of various strategies for the synthesis of vascular grafts via a suitable semi- and natural substrates, appropriate cell phenotypes, factors, and mechanical changes are inevitable [29]. Calling attention, scaffolds fabricated from biomaterials with different composition formula and mechanical properties have great potentials to regulate the development of vascular tissue.

### The application of various cell types for vascular tissue engineering

**Fig. 1:**
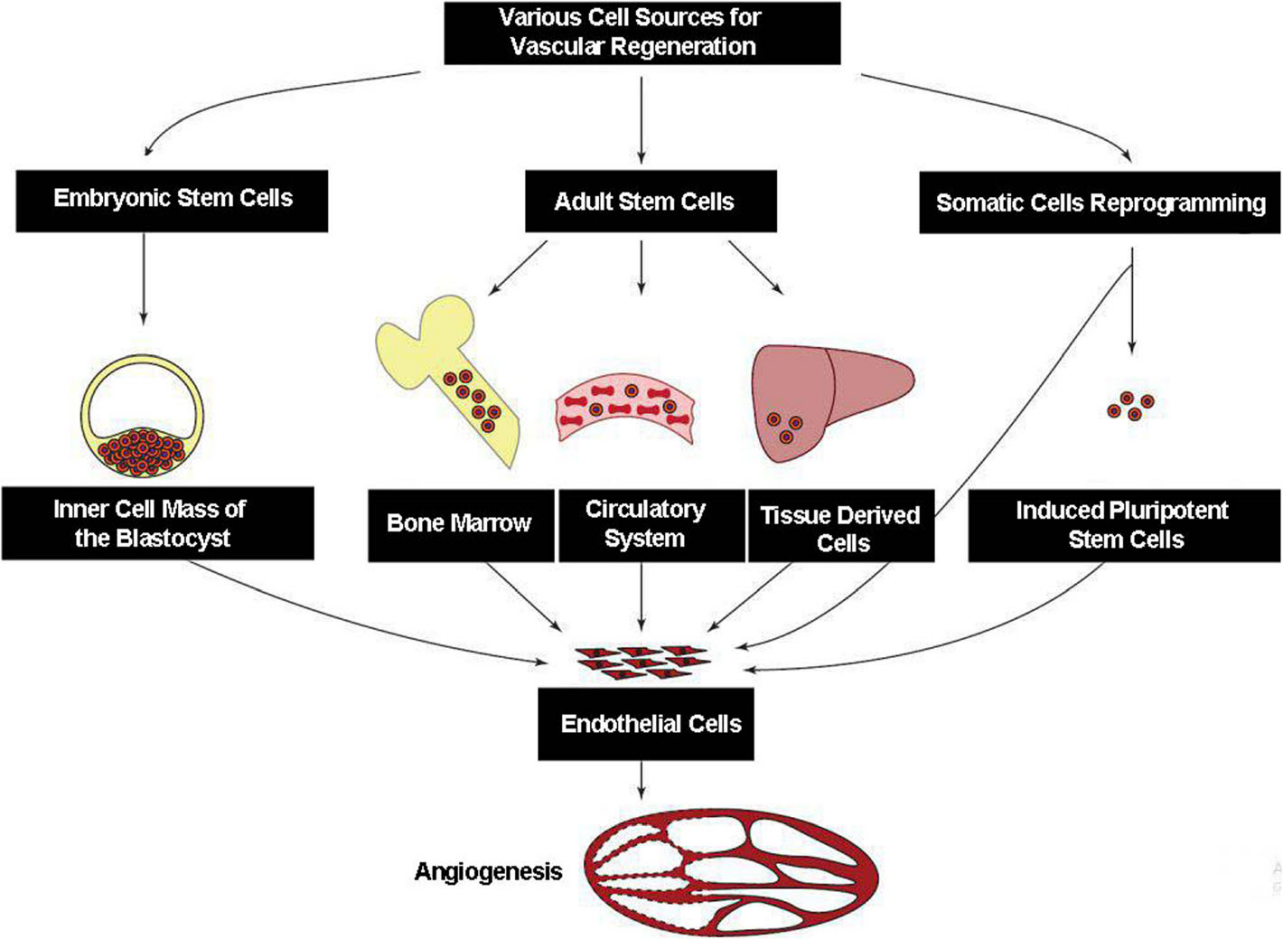
Various cell sources used at the current for vascular regeneration or induction of Angiogenesis. Many cell sources are applicable to regenerate various tissues by affecting angiogenesis and blood support

There are two main issues in vascular regenerative medicine therapy. The first one deals with the synthesis of engineered vessels and the second one is involved in the introduction of tissue constructs in promoting the growth of novel vascular networks by engaging pro-angiogenic factors. Each of these approaches seems to be effective to improve neovascularization. Due to the available cellular source in the context of angiogenesis, three main categories are routinely accepted; (**a**) somatic cells and (**b**) stem cells (either embryonic or adult cell type) and (**c**) iPSCs. In this regard, different cells from various tissues were applied to restore angiogenesis and function of ECs (Fig. 1). However, most common vascular cell types are applied for vascular regeneration include ECs, α-SMCs, pericytes, and EPCs with a strong angiogenic potential. Both allogeneic and autologous cells from mature vascular cells, including ECs and SMCs, were transplanted to subjects. Other cell types such as MSCs and iPSCs are increasingly used in numerous experiments to induce angiogenesis. Due to the lack of an immunogenic response or cell rejection upon implantation, the application of host cells is most convenient and more suitable for vascular engineering rather than the allogeneic counterpart. Compared to the progenitor cells, mature vascular cells possess a limited proliferation capacity, contributing to limited restorative effects and a low rate of pro-angiogenic outcomes [30–32]. As above-mentioned, the application of both ECs and EPCs seems to be useful for the induction of vessels formation. However, due to a high rate proliferation capacity, EPCs could promote angiogenesis efficiently compared to the mature counterpart.

### Application of ESCs in the context of angiogenesis

ESCs could be isolated from blastocysts inner cell mass having the ability to give rise to any cell type and to produce endodermal-, mesodermal-, and ectodermal-derived lineages and SCs [33]. Recent researches indicated an inherent ability of ESCs in differentiating into EC-, α-SMC- and cardiomyocyte-like cells as well [34]. During endothelial differentiation of ESCs, cell distribution of specific markers such as CD31 (PECAM), von Willebrand factor is increased. For functional analysis, different assays notably, acetylated-LDL uptake, in vitro tubulogenesis on Matrigel® and various protein substrates, staining with lectin and etc. have been introduced yet [35]. Compared to SCs, ESCs show a higher stemness feature and proliferative capacity [36]. Nonetheless, clinical applications of ESCs have some ethical issues related to the use of human embryos. Also, different immunological responses could be seen after the introduction of these cells to the target tissues. As a matter fact, it does necessitate prescribing immunosuppressive agents prior to administration of ESCs and progenies [37]. The combined regime of ESC-ECs sources has not been completely approved so far for vascular regeneration and is under early-stage trials development. Overall, it takes time to get allowable credits and translate the application of ESC for subjects with cardiovascular disease [38, 39].

### SCs

With particular success in the treatment of hematopoietic malignancies, SCs have been administrated for therapeutic use [40]. In the 1950s and 1960s, hematologists demonstrated that transplantations of bone marrow HSCs could generate a new immune system composed of many distinct functional cells. It is thought that these cells have some limitation compared to other SCs. MSCs, fibroblast-like cells, can be grown easily in in vitro condition in contrast to HSCs [31]. MSCs can be selectively differentiated into osteocyte-, adipocyte-, and chondrocyte-like cells by the modulation of various growth factors and cytokines in distinct time points [41]. A number of limitations are, however, seen in in vitro and in vivo milieu. For instance, the number of SCs are so trivial and their trans-differentiation capacity decreases by aging meanwhile the control of cell-to-cell commitment is out of control [42]. For example, a number of EPC subpopulation undergoes endothelial differentiation [43]. As a matter of fact, new approaches and strategies must be invented to dictate angiogenesis potential of each cell type. Enhancing the recruitment of progenitor cells to distinct sites, implementation of cell-to-cell crosstalk and promotion of cells alignment with vascular grafts and conduits should be determined on ongoing investigations [32].

#### iPSCs and their application

The emergence of iPSCs is considered an interesting phenomenon in regenerative medicine, paving a splendid avenue for the reconstitution of cardiac and vascular systems [44]. iPSCs are the most appropriate cell source with a great potency to giving rise to cardiomyocyte-like cells, mural cells and ECs [45]. The generation of iPSCs is achieved by the modulation of Yamanaka factors genes inside adult somatic cells, including Oct-4, Sox-2, c-Myc, and Klf-4. Cells show pluripotency properties similar to ESCs after induction of above-mentioned genes [38]. Numerous attempts have been done to investigate the vascular differentiation of iPSCs and application to human vascular research. The more recently researchers indicated that re-programming factors are sufficient to orient adult cells differentiation into the un-differentiated state. Basically, iPSCs are found to trans-differentiate into three germ layers. The therapeutic potential of iPSCs is mighty as they are patient-specific SCs hamper the immunological responses which seen in cells originated from ESCs. A plethora of experiments showed an inherent capacity of various cell types in the induction of iPSCs. The donor’s skin, fat or hair are easily accessible sources of cells [46]. Considering the lack of immunological responses, it seems that iPSCs do not have some limitation related to ESCs but the generation of iPSCs is laborious. In addition, iPSCs posse much less potential compared to the ESCs.

##### SCs delivery systems used in vascular regeneration

Researchers have so far sought to investigate the best applicable methods for SCs delivery to attain the most therapeutic effects. The primary objective in this field is to ensure an accurate homing of the transplanted cells to ischemic areas and to prolong the survival and retention of cells post-administration. More than 90% of cells are cleared from the transplantation sites during 24 h and this value is thought to reach 1% 4-week post-transplantation [47]. Because of hazardous operations, non-invasive methods are being considered as well. Novel methods and delivery approaches with inherent advantages and disadvantages having been approached for the delivery of SCs [48] (Table 2).

**Table 2:** Different cell delivery methods for the regeneration of target organs

Approach	Advantage	Limitation	Ref
Scaffolds	• Carrying cells • Delivery pro-angiogenic factors • Providing 3D condition • Having stability	• Timely degradation • Toxicity • Immune-modulatory effects	[127–130]
Stem cell priming or pretreatment	• Improve differentiation rate • Improve migration and homing rate to target tissue • Improve cell function	• Cellular senescence • Critical consideration for cell treatment	[131–136]
Exosomes	• Bio-shuttle for pro- and anti-angiogenic factors • Lack of immune-privileged capacity	• Promotes tumorgenesis • Needs to isolate and concentrated	[137–142]
Magnetic enhancement techniques	• Facilitate the cell retention rate • Control cells mobilization into target sites • Track transplanted cells in in vivo	• Provide micro-emboli for cells with small size features	[143–145]
Ultrasound techniques	• Enhance delivery of cells to target sites	• Yields cytotoxicity by promoting necrosis or apoptosis • Tissue damage such as arrhythmias, endothelium malfunction such as capillary leakage	[146–148]
Enhanced homing technique	• Enhance proliferating, migrating, and alignment of EPCs to target sites	ND*	[146, 149]
Mannitol-enhanced delivery	• Used for cell delivery through the blood-brain barrier	• Being selective for distinct cells and factors	[48, 150–152]

*Not fully determined

##### The promotion of vascularization by scaffolds

In support of angiogenesis, various scaffolding biomaterials are widely applied in tissue engineering to promote pro-angiogenic signaling pathways [46]. Scaffolds have been used for increasing the rate of transplantation outcomes and collaboration of extrinsic cells with host niche in a three-dimensional mode [49, 50]. In the case of angiogenesis, factors such as VEGF can be bound to scaffolds surface with sustained release to the surrounding tissue. Of multiple types of biomaterials, injectable scaffolds show a promising approach to promote angiogenesis. Compared to conventional surgical techniques, hydrogel scaffolds are less invasive and can be easily shaped to fill cavities and the areas of necrotic tissues [50]. Various materials with natural and synthetic structures are used to promote angiogenesis. Considering reproducibility and accessibility, synthetic materials are likely for medical translation services. The fabrication of synthetic scaffolds using different methodologies enables us to control some properties of physicochemical properties such as the elasticity and degradation rates [51]. Generally, synthetic materials are fabricated reasonable degradability at the same rate of tissue healing and growth. Natural scaffolds are commonly prepared from ECM substitutes mainly collagens, HA and fibronectin. Single purified substrates or the combined ECM proteins could be used along with decellularized ECM harboring specific cell type. The conjugation of single purified peptides and combinations of single proteins with decellularized ECM is done by using cell extract or tissue samples [52]. Due to the existence of natural analogs and distinct molecular arrangement, scaffolds formulated with ECM components are sufficient to provide anchoring-type attachment, cell growth, and trans-differentiation into various lineages. In addition, no harmful degradation products are produced which in turn enhances scaffolds integration with the body. On the other hand, control of the degradation rate, strength, and elasticity are laborious and complicate [53]. Some pitfalls of scaffolding and their applications are outlined in Table 3 [54, 55].

**Table 3:** Desirable features for biomaterials

Characteristics	Index
Biocompatibility	Rejection, Inflammation, Immune responses
3D template	To attach cells and guide growth
High surface area	Initial cell number for plating, Cell and surface interaction, Cell growth and proliferation, and cell ability to access oxygen and nutrients
Degradable	Match the rate of tissue regeneration to maintain tissue functionality
Mechanical stretching	Consistency against to biological forces
Enrichment with growth factors cocktail	Support the cells in synthesizing tissue-specific extracellular matrix components and growth factors required for healthy tissue growth
Stability	To prevent cell cytotoxicity without alteration in physical values
Serve as a barrier	To elicit a barrier between luminal and body cavity
Support the induction of vascular structure	Muscle tissue regeneration in aligned pattern to promote appropriate innervation and vascularization

During the last decades, demands for vascular transplantation have been raised after the initiation of cardiovascular disease while rejection could be seen just after the transplantation. Lack of proper donors and failure in the process of surgical operations has led to increasing researchers in the field of vessel engineering and the production of the vascular grafts [56]. A scaffold can play an important role in tissue engineering and restoration. In recent decades, the use of biomaterials similar to vessels structure has been considered extensively by different authorities. Engineered vascular grafts require multiple factors to facilitate compatibility with natural veins and increase production functionality in vivo. Some of these features are considered for the development of vascular prosthesis. Characteristics such as proper porosity, a low rate of thrombogenicity and immunogenicity with minimum harmful effects on blood cells, enzymes, and plasma proteins should be notified in the fabrication of tissue-engineered vascular structures [57, 58]. Additionally, biodegradation is one of the specific requirements for tissue-engineered grafts and determined by scaffold components and mechanical properties [59]. Cell attachment to scaffold moieties is essential for the activity of cells over a time thus substances in vessel grafts should have sufficient cell adhesion properties. The existence of bioactive moieties in scaffolds promotes the juxtacrine connection of ECs with pericytes and cells expressing factor α-SMA [60]. Regarding the prevalence of cardiovascular disease and a higher mortality rate among old patients, investigations in heart and vessels transplantation techniques, design and use of appropriate biomaterials with the ability to preserve cell activity are highly needed.

### Biomaterials

The term of the scaffold expresses a temporary structure that causes tissue growth and formation in the biological environment by providing a 3D environment. Creation of small biological environment will result in cell growth, differentiation and acquisition of specific phenotype. Scaffold materials can be classified into both natural and synthetic categories. The natural ECM substrates with specific particle diameter are highly dynamic and have complex interplay with cells. Scaffolds made of natural materials contain specific ligands for cellular connectivity, cell migration, and various growth factors to achieve a strong restoration rate. Based on the tissue compatibility and stiffness rate, various materials are commonly used for the restoration of specific organs (Table 4). As a result, the selection of materials depends on the target tissue consistency and defect severity [61–63]. From a certain point of view, scaffolds are biological materials with clues for the promotion of cell growth and tissue regeneration. In addition, enhancing the angiogenic potential of host tissue allows a higher degree of control on cell behavior after transplantation. Commensurate with these comments, scaffolds have the potential to harbor distinct growth factors, provide moieties for cell attachment and develop the 3D condition for cell-to-cell communication is highly recommended. These features enable scaffolds to efficiently induce angiogenesis and cell adaptation after transplantation into target sites.

**Table 4:** Advantage and limitation of different biomaterials in tissue-engineered approaches

Scaffold	Advantage	Limitation	Ref
Collagen	• Highly distensible and pressure sensitive • Having well-organized pattern • Resistant to high strain and decrease the permeability of the vascular structure	• Thrombogenic potential and activation of the coagulation cascade • Enhanced risk of immunogenicity • The high cost of pure collagen	[153]
Elastin	• Suitable for high porous structures with a small diameter • Enhanced the proliferative capacity of ECs • Enhanced cell dynamics and rearrangement of collagen after tension	• Solubilizing difficulty • Inefficient mixing with other polymeric materials	[65]
Matrigel	• Comparability to extracellular matrix	• Minimally invasive • Degradation time	[154]
Fibrin	Suitable for delivery of thrombin, fibrinogen and coagulation factors	• Structural weakness • Suitable for the fabrication of synthetic transplants (PEG, PLGA)	[69]
Alginate	Used commonly polymer for encapsulation	Control of size	[74]
Chitosan	Easily form polyelectrolyte complexes with other polyanions	Poor mechanical property	[75, 155, 156]
Agarose	Available as agarose, is gelatinous and has sol-gel transition based on temperatures	A wide range of commercially available agarose	[157]
HA	low HAs enhances the proliferation and migration of ECs	The high molecular HAs inhibits angiogenesis	[158]

### Collagen

Features of collagen in scaffolds allow fabricating products with graded elastic stiffness. Despite these advantages, collagen breakdown products in vivo may cause to release of thrombogenic materials such as amino acids, resulting in an increase of immunogenic reactions. However, the most common problems with using collagen scaffolds are the high cost of earning a pure solution. Collagen is the most abundant protein in ECM synthesized by fibroblasts and bone osteoblasts [63, 64]. Due to optimal stiffness property, collagen could be considered as an appropriate substrate for the synthesis of engineered vessel grafts. However, cautions must be taken related to the release of degradation products into systemic circulation.

### Elastin

The shape and elasticity of blood vessels depend on the amount of elastin substrate. Scaffolds with small porosities made of the combination of elastin and collagen are suitable for fabricating small-diameter vessels. Compared to collagen, the insoluble form of elastin has higher strain recovery [65]. The proliferative potential of ECs was found to enhance by the mixture of elastin and collagen gels. Notably, scaffolds with elastin origin could enhance cell activity, rearrangement, stability, and mobility. In some experiments, it was demonstrated that the combination of elastin with other polymers could yield more solubilizing and transparent mixture compared to elastin scaffolds [66]. It seems that elastin could be applied for fabrication of small-diameter vascular grafts and its combination with other substrates is useful for the synthesis of large-diameter vascular units.

### Fibrin

Fibrin can be isolated from patient blood and mix with scaffolds for therapy aims [67, 68]. Fibrin is a type of protein with a high density and potential for survival of transplanted cells. The application of fibrin in scaffolds is a traditional method for delivery of thrombogenic factors, bone marrow mononuclear cells and various cytokines such as bFGF [69]. In spite of the benefits, fibrin has some drawbacks, for example, the extra addition of fibrin causes structural weakness [70]. It is hypothesized that fibrin has a great potential to transfer growth factors and distinct cells to the target sites. Because of lytic susceptibility and thrombogenic activity, the application of fibrin has been limited in the structure of engineered vessels grafts.

### Alginate

The most frequently used polymer for encapsulation of therapeutic agents is alginate. Alginate is the most studied material for encapsulation of living cells from different sources. Due to a unique feature and molecular structure, alginate has been extensively applied to increase angiogenesis and endothelial differentiation after combination with factors such as VEGF and bFGF. Despite an unregulated hemangioma formation and vascular leakage, small doses of alginate could bring therapeutic outcome without any complications. For example, alginate encapsulation of transplanted cells containing heparinized group provides prolonged sustained release of growth factors in infarct areas [71–74]. However, alginate has an excessive negative charge, limiting the cell attachment and alignment. The combination of alginate with natural substrates could circumvent these pitfalls and limitations. In line with these claims, the application of alginate-based scaffolds must be considered after the completion of further investigations.

### Chitosan

Chitosan, a type of polysaccharide termed as chitin, is extracted from exoskeleton in many species. Chitosan could be combined with other polyanions because of unique molecular properties. It was elucidated that the combination of chitosan/alginate (alginate bead) with poly-L-Lysine improves chitosan biodegradability and biocompatibility by changes in pH and solubility. In this regard, various structural modifications can also be chemically done on chitosan in favor of tissue engineering [51, 75].

### Scaffold-based miRNA therapy

A large number of experiments showed that miRNAs can change the dynamic growth of SCs and somatic cells. These features were further determined by monitoring the expression and inhibition of specific miRNAs by complementary sequences [76, 77].

### miRNA replacement therapy

Differentiation is a process that causes to change the level of miRNA in stem cells and various cell types. In many surveys, this strategy is used for the orientation large-scale differentiation of progenitor cells by modulating the level of distinct miRNAs. Modulations are performed based on the application of miRNAs with two approaches; (**I**) Delivery of target miRNA that has the same sequence with double strand oligonucleotides, mimicking the same structure with the ability to enter miRISC complex and thereby intensify the content of target genes. (**II**) Introduction of specific genetic materials (miRNAs) inhibits the function of target genes. This model has a unique potential for sustained release of miRNA, however, a disadvantage such as off-target effects should not be neglected [78, 79].

### miRNA inhibition therapy

The aim of miRNA inhibition therapy is to stop or decrease certain miRNA expression. In this regard, some methodologies are applied to destruct miRISC complex. The most direct way for inhibition between miRISC and miRNA is the application of AMO [80, 81]. However, this strategy may result in unwanted side effects because a single miRNA can regulate different genes. Therefore, miRNA masks are developed to minimize the off-target effects and selectively block specific mRNA pathway, contributing to the inhibition of specific protein [82].

### miRNA in angiogenesis

miRNA is one of the most common factors for the induction/inhibition of angiogenesis. For instance, the overexpression of miRNA-503 in ECs leads to migration, proliferation, stimulation, and cell division via the modulation of cyclin E. In another study, cardiac ECs expressing miRNA-24 showed profound changes in the transcription level of ATA2, P21 kinase PAK4, apoptosis, and cell sprouting rate. It was showed that miRNA-24 could abort myocardial function and angiogenesis rate in mouse cardiac cells. Despite the benefits of angiogenesis, there is a close relationship between vascularity and tumor expansion. For example, a cluster of miRNAs (miRNA-17, −18a, −2a, −19b-1, and − 17-92) increase angiogenesis in both of in vivo and in vitro conditions. Nevertheless, miRNA application is enthusiastically welcomed for the induction of angiogenesis rather than repression [83–86]. Commensurate with these comments, it is logical to assume that two approaches, miRNA replacement, and induction, could be applied in the field of tissue-engineered vascular grafts and angiogenesis by modification of genetic elements in the target cells.

### miRNA delivery systems

**Fig. 2:**
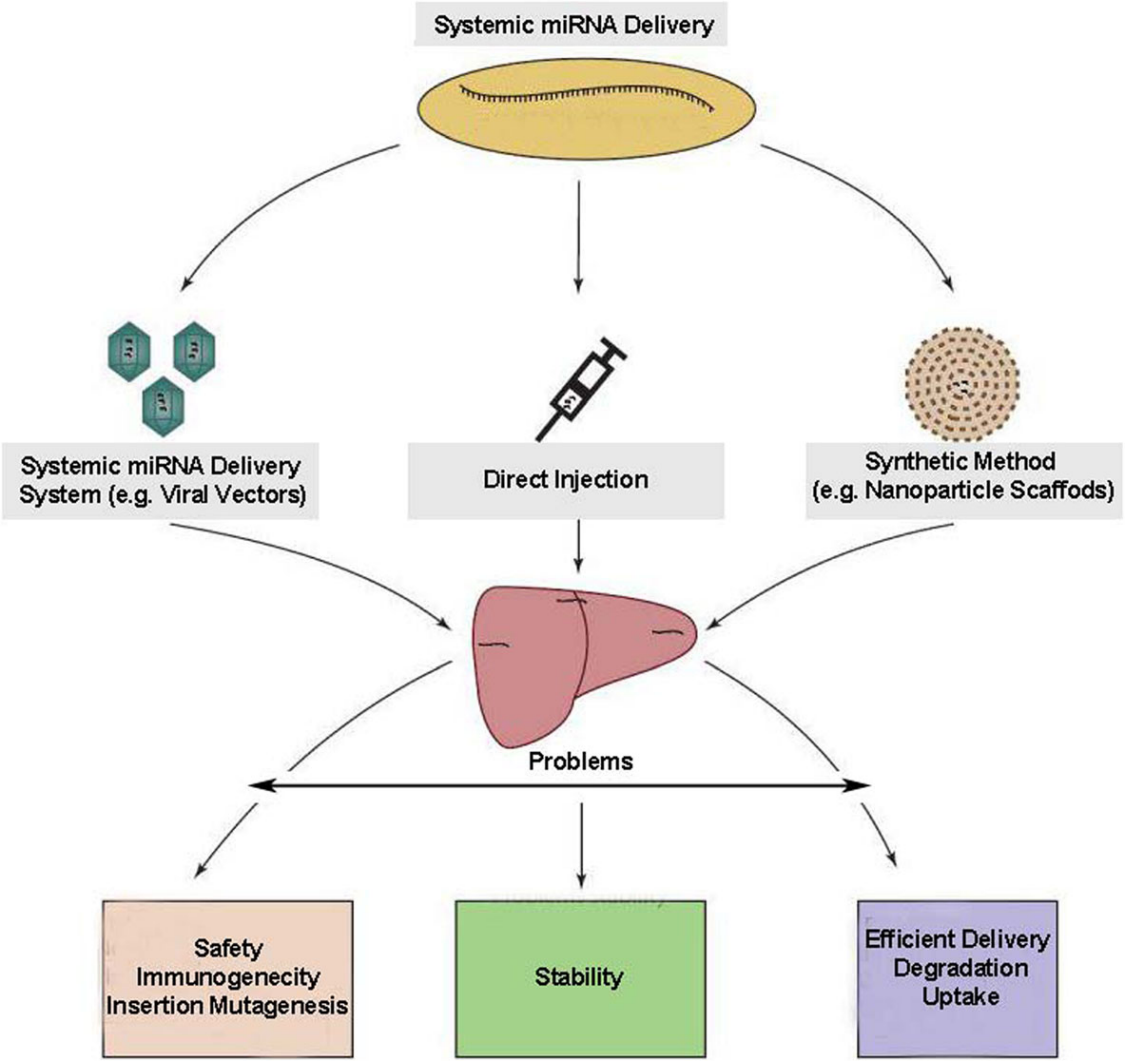
miRNA delivery systems in the field of tissue engineering

Different miRNA delivery systems have been established in the field of regenerative medicine (Fig. 2). Commonly methods for miRNA delivery are direct injection and the application of viral/non-viral vectors.

#### Systemic miRNA delivery systems

Viral vectors are another method for delivery of specific genomic pool to a large number of cells. Due to the susceptibility to degradation by RNase, the main pitfall of miRNA therapy pertains to short shelf-life period. Additionally, miRNA concentration decreases with the progression of cell division. Therefore, they only exert a transient silencing effect. The main barrier for clinical translation of the viral vector is a safety problem that was caused by immunogenicity and insertion mutagenesis. Different viral vectors have been approved yet, however, each of them can have own limitations and advantages. In the biological field, most popular viral vectors are adenoviral and AAV, lentiviral and retroviral vectors. AAV is the most commonly used between other viral vectors. This viral-vector is a non-enveloped virus that needs adenovirus for completing own amplification. This vector has single strand DNA with 4.7Kb and 12 primate serotypes (AAV1–12). On the other hand, AAV with small size and non-pathogenicity for a human is an appropriate selection for delivery of miRNAs. Retroviruses are commonly used for cellular reprogramming and genetic manipulation [80, 86, 87].

#### Direct injection

Direct systemic injection is the simplest method for delivery of miRNAs but the main problem for this way is short-term stability. In support of this notion, miRNAs are very sensitive and can be rapidly cleared by kidneys. They also attach to plasma proteins and/or degraded by nucleases enzymes in serum. Nonetheless, it has proven that the application of some miRNA inhibitors increases miRNAs stability and function following intravenous and subcutaneous injections. For instance, intravenous injection of LNA-anti-miRNA showed the silencing of miRNA-122 in the liver and the decrease of plasma cholesterol. It has also been indicated that a single bolus injection of LNA-anti-miRNA is active for several weeks [88, 89]. In some circumstances, the direct injection of miRNAs could provoke immune response elements and thereby some strategies must be considered to limit unwanted immune reactions.

#### Exosomal delivery

Exosomes are nano-sized particles, ranging from 40 to 150 nm, present in body fluids. They encompass specific biomolecules and specific genetic modulators that control numerous biological activities [90]. Considering the potent ability in reciprocal communication, exosomes could maintain steady-state crosstalk between different cells by using autocrine, paracrine and juxtacrine pathways. The active role of exosomes was previously proved on blood vessels development and progression [91]. Recent evidence suggests the existence of specific miRNAs inside exosomes with the angiogenic potential in the target cells by the induction of VEGF and different signaling pathways [92, 93]. In contrast, exosomes could harbor some miRNAs with anti-angiogenesis properties [94] (Table 5). It was revealed that the conjugation of exosomes with tricalcium phosphate scaffolds promoted osteogenic differentiation of bone marrow MSCs through the activation of PI3K/Akt signaling axis [95]. By increasing the concentration of transplanted exosomes in tricalcium phosphate-based scaffolds, there was a profound bone formation and angiogenesis rate in the rat model of bone defects [96]. These features indicated the potential of exosomes as bio-shuttles in modulating the vascularization rate after being loaded on distinct scaffolds.

**Table 5:** The existence of various miRNAs with pro- and anti-angiogenesis capacity

miRNA	Function	Ref
miRNA-17-92	Promotes angiogenesis by modulation of connective tissue growth factor, thrombospondin-1, and integrin α5	[159]
miRNA-92a	Has a dual pro- and anti-angiogenic role	[160]
miRNA-21	Increases VEGF level and promotes angiogenesis through a STAT3-dependent mechanism	[161]
miRNA-494	Suppresses PTEN and activates Akt/eNOS pathway	[162]
miR-135b in exosomes from hypoxic multiple myeloma cells	Reduces the expression of FIH-1 and increased activity of HIF-1α	[163]
miR-125a	Promotes angiogenesis by inhibiting DLL-4	[92]

#### Synthetic methods

Non-viral-based approaches for genetic material delivery have attracted the attention of a bulk of authorities over the last decades. In the late 1990s, the term gene-activated matrixes emerged and the first report documented collagen-based scaffolds used to deliver galactosidase-based pDNA for the acceleration of bone formation. At the present time, numerous synthetic delivery systems are used with miRNAs as follows;

(a) lipid-based; (b) polyethyleneimine (PEI)-based; (c) dendrimer-based; (d) poly (a-hydroxy acid) polymers (in nano-particle or scaffold form); (e) fabrication of biopolymers as particles such as chitosan and protamine, atelocollagen, and protein translocation domain-derived peptides or scaffolds, (f) inorganic nanoparticles (gold, silica-based, or magnetic) and scaffolds. In the majority of methods, the synthesis is governed by self-assembling of synthetic materials such as lipids (liposomes), unprocessed polymers (dendrimers), or functionalized polymers with active sites. Indeed, it is such self-assembled nature that offers considerable superiorities over viral-based methods with potential for controlling molecular composition, targeted ligand-receptor attachment, tolerance of large (multiple plasmids) cargo sizes, disassembly and release of payloads, simplified manufacturing, modification, scale up, ease of analysis and quality control, and low immunogenicity rate [97]. These synthetic systems possess similar efficacy to viral-based methods in vitro. The use of synthetic systems in in vivo condition is increasing as well; however, challenges remain in terms of efficacy via intravenous versus local injection, sufficient delivery of miRNAs to the site of injury without degradation or nonspecific binding, appropriate uptake by the appropriate cell type within complex tissues, and, thereafter, persistence of gene expression or inhibition in favor of regeneration [98, 99]. Non-viral carriers seem to be more effective compared to viral counterparts, however, the lack of specificity to target sites and uncontrolled bio-distribution limit the use of non-viral approaches.

### Growth factor incorporation into scaffolds

Various growth factors could concurrently be combined with scaffolds to facilitate regeneration rate. In general, scaffolds-coated with growth factors could promote the introduction of these molecules to target sites, expending the rate of recovery. Due to an inherent kinetics and different features controlling the release of growth factors, the more fundamental experiments are needed to address underlying mechanisms. Of note, the protein structure and function must not be changed during the procedure. VEGF is a peptide growth factor that recently coated on the PLA-based scaffold for controlling angiogenic signals [100]. The sustained release and active dynamic of VEGF were confirmed by several techniques in vitro in HUVEC assay and in vivo condition such as chick allantoic membrane. It was showed that VEGF has a unique role for vascularization in PLA-based scaffolds. Different growth factors can be loaded to the surface of scaffolds via interaction with chemical groups of drug and proteins. In support of this idea, such scaffolds are synthesized to mimic in vivo microenvironment with the ability of growth and differentiation for human SCs. Prior to enrollment in tissue engineering procedures, the function of transplants can be improved by using multiple growth factors with different formulas, offering a wonderful way to control tissue regeneration. However, the release of specific protein must be elucidated on distinct tissues to specific cells in the context of target tissue [101]. In spite of an enhanced angiogenesis rate induced by the mixture of scaffolds-growth factors, normal kinetics and appropriate sustained release of each factor must be calculated in in vivo condition.

### Effect of biomaterials on the intracellular angiogenesis signaling pathway

**Fig. 3:**
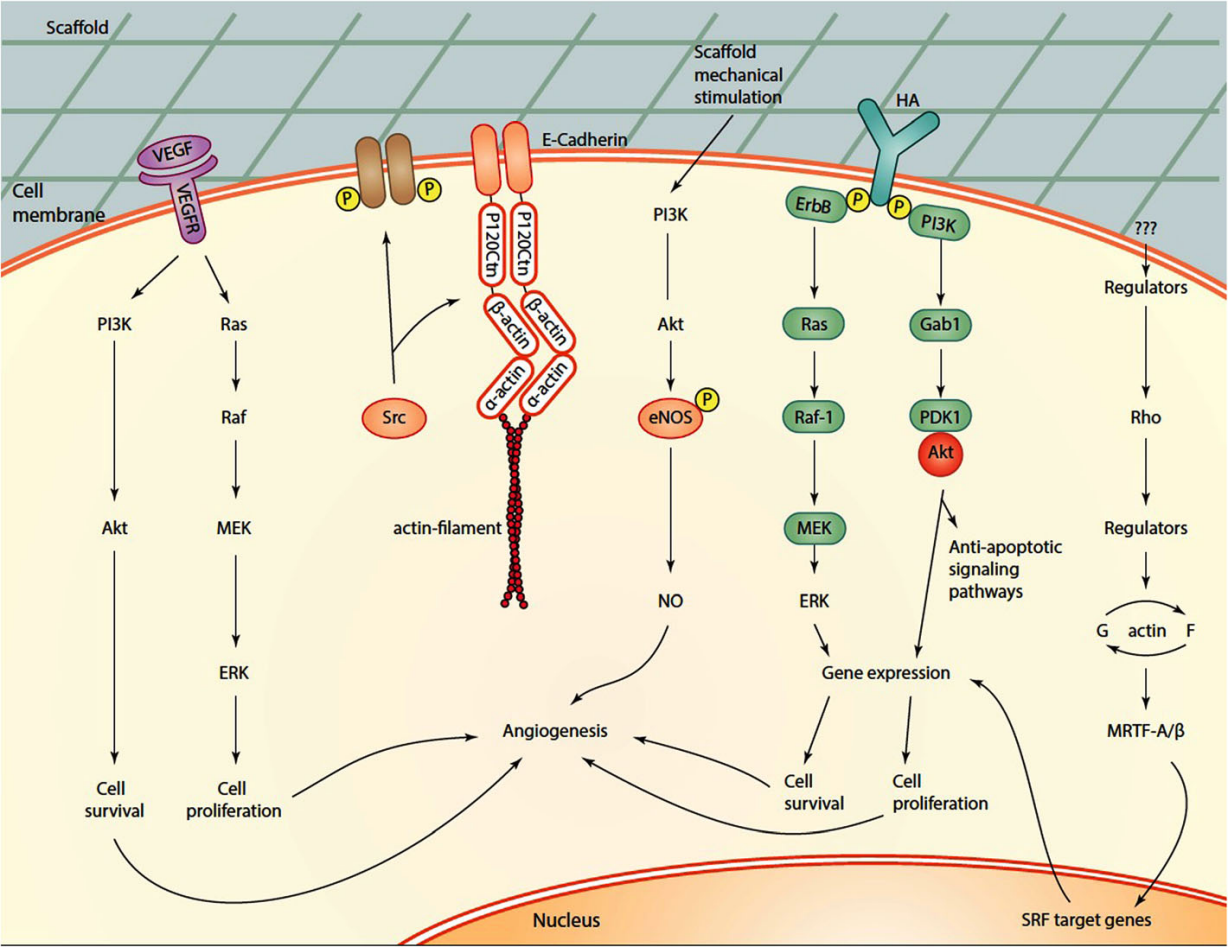
Effect of different of biomaterials on intracellular angiogenesis signaling pathways. VEGFR-2, E-cadherin and CD44 could be initiated after juxtaposition to different scaffolds. Signaling pathways effectors such as Akt and Raf and Erk are stimulated during angiogenesis potential. HA: Hyaluronic acid

It is believed that the changes in the physicochemical and morphological properties of cells on different scaffolds could modulate the angiogenesis potential [102] (Fig. 3). The mutual crosstalk between cells and surrounding scaffolds via adhesive molecules such as laminin, fibronectin, vitronectin, tenascin, and hydrophilic proteoglycans can initiate specific signaling, termed as external transduction [103]. On the other hand, the cell could attach to surfaces by expressing integrins, immunoglobulin superfamily, cadherins, selectins, and other adhesive molecules [104]. Using juxtacrine interaction of cell receptors with cognate motifs in scaffolds, a plethora of intracellular biochemical reactions would be ignited, resulting in the modulation of cells phenotype, motility and migration, dynamic cell growth and genes expression profile. In addition to the maintenance of cell-matrix interaction, it seems that the cell-to-cell connection is improved as well [105].

The juxtaposition of each cell with scaffold chemical groups is done through the activation of surface mechanoreceptors and thereby a reciprocal bridge is formed. This attachment provokes contractile cytoskeletal agents with the collaboration of focal adhesion complexes [106]. The cytoskeletal adaptation was reported in ECs cultured in a denaturated collagen matrix by re-arranging actin filament and distribution of focal adhesion proteins such as Src-dependent signaling pathway [107].

The scaffold enriched by beta-tricalcium phosphate has been found to promoted angiogenesis in HUVECs by engaging the PI3K/Akt/eNOS axis [108]. In addition to the chemical composition of scaffolds, the 3D alignment and microstructure could stimulate and/or inhibit the normal dynamic of cells. By adding fibrin to collagen-based scaffolds, the basal metabolic activity of cells showed to be altered which was evident by an enhanced proliferation rate. The migration of enclosed cells was increased by the induction of MMP-2 and -9 [109]. It was shown that fibrin could attach to cell surface integrin α_v_β_3_ and improve the interaction of circulating leukocytes with ECs via the modulation of VE-cadherin [110]. The promotion of cell recruitment and orientated differentiation could yield in better regeneration capacity [111]. Matthew and colleagues previously stated that the enrichment of collagen scaffold with HA had a prominent effect on the cell recruitment and differentiation into endothelial and osteoblast lineages [112]. Previously, the positive effect of HA was indicated on the dynamic of angiogenesis mediated by CD44 and protein kinase C (δ). Fibrin was also found to activate plasminogen activator-inhibitor-1, TGF-β receptor I and Erk [113]. Experiments revealed that the intra-fibrillar silicified collagen forced monocytes to secret SDF-1α, TGF-β1, VEGFA, and PDGF-BB which are a key regulator in the initiation of angiogenesis [114]. Addition of ionized calcium with PLA and on bioactive glass G5 promoted angiogenesis by stimulating factors GATA2, TFII-I, and NF-кB. The expression of VEGFR-2, as a tyrosine kinase-related receptors, was also induced as well [115]. The use of suitable scaffolds with appropriate physicochemical stability and combination with various factors and ECM components could re-organize cells alignment in the favor of vascularization and engineered vascular grafts.

### Conclusions and future perspectives

In accordance with a great body of previous studies and what is highlighted in the current review article, angiogenesis is the main target and reliable mean to increase the efficiency of tissue regeneration by cell transplantation, gene therapy, and factor release. Based on target tissues, inherent advantages and limitations of each delivery method must be considered. Choosing distinct cell type, selection of scaffolds and carriers fabricated by different biomaterials, and orientation of cells to vascular cells using growth factors and genetic manipulation seem pivotal to accelerate the vascularization rate. It seems that different scaffolds could influence the rate of angiogenesis via regulating cell morphology and alignment inside the matrices. Calculation of appropriate initial cell number for transplantation, route of administration (either local or systemic), contents and growth factor formulation along with transient and/or permanent genetic modification are also important. In some cases, cell-free strategies could also eliminate the need for simultaneous application of cells with growth factors. As a matter of fact, application and invention of novel strategies with the capability to preserve factors for long periods with a sustained release activity must be at the center of attention. Exosomes, as cell byproducts encompassing a large number of factors, having a high stability could be introduced as angiogenic bio-shuttles with various scaffolds without any unpredictable complications. In addition to the composition and structure of scaffolds, the bioavailability, biodegradability, and route of administration must be detected related to distinct tissue type.

## Abbreviations

AAV: Adeno-associated virus
AMO: anti-miRNA oligonucleotide
Ang-2: Angiopoietin-2
bFGF: Basic fibroblast growth factor
CECs: Circulating endothelial cells
CECs: Circulating endothelial cells
DDL-4: Delta ligand 4
ECM: Extracellular matrix
EPCs: Endothelial progenitor cells
FIH-1: Factor inhibiting HIF-1
HA: Hyaluronic acid
HSCs: Hematopoietic stem cells
HUVECs: Human umbilical vein endothelial cells
iPSCs: Induced pluripotent stem cells
miRISC: miRNA-induced-silencing-complex
miRNA: microRNA
MMP-2 and -9: Matrix metalloproteinase-2 and -9
MSCs: Mesenchymal stem cells
PDGF-BB: Platelet-derived growth factor
PECAM: Platelet endothelial cell adhesion molecule
PEI: Polyethylenimine
PLA: Poly(lactic acid)
SCs: Adult stem cells
SDF-1α: Stromal cell-derived factor 1
SMC: Structural maintenance of chromosomes
STAT3: Signal transducer and activator of transcription 3
TGF-β receptor I: Transforming growth factor β receptor I
VE-cadherin: Vascular endothelial cadherin
VEGF: Vascular endothelial growth factor
VEGFR2: Vascular endothelial growth factor receptor 2
α-SMCs: alpha smooth muscle cells
